# Prior subclinical histoplasmosis revealed in Nigeria using histoplasmin skin testing

**DOI:** 10.1371/journal.pone.0196224

**Published:** 2018-05-09

**Authors:** Rita O. Oladele, Conchita Toriello, Folasade T. Ogunsola, Olusola O. Ayanlowo, Philip Foden, Adetona S. Fayemiwo, Iriagbonse I. Osaigbovo, Anthony A. Iwuafor, Shuwaram Shettima, Halimat A. Ekundayo, Malcolm D. Richardson, David W. Denning

**Affiliations:** 1 Department of Medical Microbiology & Parasitology, College of Medicine, University of Lagos, Lagos, Nigeria; 2 Faculty of Biology, Medicine and Health, The University of Manchester, Manchester, United Kingdom; 3 Global Action Fund for Fungal Infections, Geneva, Switzerland; 4 Facultad de Medicina, Universidad Nacional Autónoma de México, Mexico City, Mexico; 5 Department of Medicine, College of Medicine, University of Lagos, Lagos, Nigeria; 6 Medical Statistics Department, University Hospital of South Manchester, Manchester, United Kingdom; 7 Department of Medical Microbiology & Parasitology, College of Medicine, University of Ibadan, Ibadan, Nigeria; 8 Department of Medical Microbiology, University of Benin Teaching Hospital, Benin City, Nigeria; 9 Department of Medical Microbiology & Parasitology, College of Medical sciences, University of Calabar, Calabar, Nigeria; 10 Department of Medical Microbiology, Federal Medical Centre Yola, Yola, Nigeria; 11 Department of Medical Microbiology, General Hospital Ilorin, Ilorin, Nigeria; 12 Mycology Reference Centre Manchester, University Hospital of South Manchester, Manchester, United Kingdom; 13 National Aspergillosis Centre, University Hospital of South Manchester, Manchester, United Kingdom; University of Ottawa, CANADA

## Abstract

**Objectives:**

Disseminated histoplasmosis is an AIDS-defining illness. Histoplasmosis is commonly misdiagnosed as tuberculosis. Nigeria has the second highest number of people living with HIV/AIDS in Africa. The present study was carried out to investigate the prevalence of skin sensitivity amongst Nigerians to histoplasmin.

**Design:**

A cross-sectional study was conducted in six centres across five geopolitical zones of Nigeria.

**Methods:**

We recruited both healthy non-HIV and HIV-positive adults with CD4 count ≥ 350 cells/mm^3^ regardless of their ART status from March to May 2017. Skin tests were performed intradermally; induration ≥5 mm were considered to be histoplasmin positive.

**Results:**

750 participants were recruited from Lagos (n = 52), Yola (n = 156), Ilorin (n = 125), Calabar (n = 120), Ibadan (n = 202) and Benin (n = 95). 467 (62.3%) were HIV negative, 247 (32.9%) were HIV positive and 36 (4.8%) did not know their HIV status. A total of 32/735 (4.4%) participants had a positive skin test. Study centre (p<0.001), education (p = 0.002) and age (p = 0.005) appeared to be significantly associated with positive skin reactivity at the 0.5% significance level, while sex (p = 0.031) and occupation (p = 0.031) would have been significant at the 5% significance level. Males had a higher rate of reactivity than females (p = 0.031, 7% vs 3%). The highest positive rates were recorded from Benin City (13/86 (15%)) and Calabar (7/120 (6%)) and no positives were recorded in Lagos (p<0.001). HIV status was not statistically significant (p = 0.70).

**Conclusion:**

Histoplasmosis diagnostics should be included in the Nigerian HIV guidelines. Epidemiological vigilance of progressive disseminated histoplasmosis should be considered by local health authorities.

## Introduction

Histoplasmosis is considered a disease of worldwide distribution, with hyperendemic areas. The presence of the pathogen and clinical disease are described in large geographical areas, including most of Asia and Africa [1]. Histoplasmosis is caused by *Histoplasma capsulatum*, a thermally dimorphic ascomycete. Skin-test surveys using histoplasmin as a reagent, which is similar to the tuberculin test, have shown evidence of variable exposure throughout Central America and parts of South America as well as Puerto Rico, Dominica, and Mexico in addition to the central USA with almost no skin-test positivity in Europe [2]. In Nigeria, a higher prevalence of skin test reactivity (≈35%) was found in a rural population, especially among farmers, local traders, and cave guides [3]and this was prior to the outbreak of the HIV epidemic. In Africa, surveys of histoplasmin skin sensitivity have demonstrated positivity rates ranging from 0.0% to 28%, [4–12] and cross reactivity has been demonstrated between *Histoplasma capsulatum var capsulatum* (Hcc) and *Histoplasma capsulatum var duboisii* (Hcd) in Nigeria [7].

Given that disseminated histoplasmosis is an AIDS-defining illness with a high mortality [13], and that many cases of histoplasmosis have been reported in Nigeria [14,15], it is important to consider whether exposure is or is not common in different locations in Nigeria. Nigeria with a HIV prevalence of 3.7% and an estimated population of 170 million people has the second highest number of people living with HIV in Africa. In histoplasmosis endemic areas, histoplasmosis is a primary consideration in human immunodeficiency virus (HIV)–infected patients with suspected tuberculosis [16]; WHO estimated the incidence of TB in Nigeria as 322 per 100,000 population with only 15% of the total burden of the disease in the country being documented with Genexpert and/or acid alcohol fast bacilli smear in 2015 [17]. Data on histoplasmin skin sensitivity screening is more than 3 decades old in Nigeria and was obtained before the HIV epidemic it has not been repeated again since [18]. Documentation of histoplasma exposure will assist in tailoring diagnostic testing, especially in HIV-infected patients.

## Methodology

We performed a detailed literature search of published reports of histoplasmosis in Nigerians between the periods of 1969 to Jan 2017. The literature search for publications was done using Pubmed, Web of Science, Google Scholar, African Journals Online (AJOL), Africa-Wide: NiPAD, CINAHL (accessed via EBSCO Host) databases, and grey literature to identify all published papers regarding the topic. Articles published in other languages (e.g., French, German, and Portuguese) were considered if they were cited in any of the databases searched. The main search comprised individual searches using detailed medical subject heading (MeSH) terms for histoplasmosis, Nigeria, and HIV/AIDS combined with terms relevant to histoplasmosis, including broad terms such as ‘case report’, ‘diagnosis’ and ‘management’. The Boolean operator ‘AND’ and ‘OR’ were used to combine and narrow the searches. The references in all relevant papers were reviewed for additional publications that may not have been cited elsewhere (‘snow balling’), as well as our own paper files. One hundred and twenty four cases of histoplasmosis were identified between 1969 and 2017 (see S1 File). Based on these findings, we chose three areas with published case reports (Ibadan, Ilorin and Calabar) and 3 with no published cases (Yola, Lagos and Benin) were selected; this was to ensure representation of the geopolitical zones in Nigeria. Six centers were chosen; they reflected five out of the six geopolitical zones in Nigeria. (South Eastern Nigeria- Calabar; Northern Nigeria- Yola; North central Nigeria—Ilorin; South southern Nigeria—Benin City and South Western Nigeria—Ibadan; Lagos); the sixth zone had on-going militant insurgency. Both HIV infected patients attending a PEPFAR clinic with CD4 count ≥350 cells/mm regardless of their ART status and healthy persons from the community (whose HIV status are unknown) were recruited consecutively. CD4 count cut-off value of 350 cells/mm^3^ was chosen because patients with lower levels may not elicit adequate immune response, which may result in false negative histoplasmin skin sensitivity. Ethical approval was obtained from the institutional Ethics Committee of the study sites’ Health Management Boards; Calabar (RP/REC/2016/444), Ibadan (UI/EC/16/0432), Benin (ADM/E22/A/VOL.VII/1468), Yola (FMCY/SUB/96N/T/X), Lagos (NHREC:19/12.2008a; ADM/DCST/HREC/662) and Ilorin (MOH/KS/EC/777/115). Informed verbal and written consent was obtained from each study participant after adequate explanation of the study and its objectives. Participants with a previous medical history of histoplasmosis and those who did not return for skin test reading were excluded.

### Data collection

A structured questionnaire was used to obtain data relating to socio-economic and demographic data, and risk factors associated with *Histoplasma* infection from consenting participants. The risk factors included; recent (up to a year before) or past (more than a year ago) activities involving soil (gardening, civil construction or agriculture) or visits to farms or caves, or the presence of birds or bats in the home or neighborhood; and travel history. Clinical and laboratory data, such as time of HIV diagnosis, CD4 cell count, ART and antifungal therapy was obtained from the HIV-infected patients’ records.

#### Antigen preparation

The *Histoplasma* antigen was prepared by Dr Conchita Toriello lab similar to prior studies of histoplasmin [16, 17] at Universidad Nacional Autónoma de México. In brief, *Histoplasma capsulatum* EH53 strain which is registered in the World Data Centre for Microorganisms (WDCM) database under the acronym LIH-UNAM WDCM817, was obtained from the fungal collection of the Fungal Immunology Laboratory of the Department of Microbiology-Parasitology, Faculty of Medicine, National Autonomous University of Mexico (UNAM). Strains were maintained in Sabouraud dextrose agar (Bioxon, Mexico) at 4°C and inoculated in mice to check and regain virulence. Three-week-old mycelia from solid Sabouraud dextrose cultures at 28°C were inoculated into 1L Erlenmeyer flasks with 250 ml of Smith’s asparagine medium [19]. They were maintained in static cultures at 28°C for 2 months. Cultures were checked for their characteristic morphology. Two month-old cultures of the organism were killed with addition of 0.05% thimerosal (final concentration) at 28°C for one week. Killing was routinely checked by culture of treated mycelia in Sabouraud dextrose agar. Killed cultures were filtered through a 0.45um Millipore membrane; the filtrate was dialyzed and concentrated 10-fold using an Amicon ultrafiltration system with a PM-10 membrane (Amicon Corp., Lexington, MA). Each filtrate containing crude antigens, including histoplasmin was subjected to further treatment by phenol extraction, ethyl alcohol precipitation, and deproteinization by pronase and Sevag [19]. Purified antigens (PPC-histo) were the products of this last procedure. The purified antigens were adjusted to 2.5mg carbohydrates/mL and 0.5–1.0mg protein/mL [20]. There is documented evidence of cross reactivity between *H*.*capsulatum* and *H*.*duboisii* in our environment [7, 18].

#### Skin testing

Skin tests were performed by intradermally injecting 0.1 mL of histoplasmin antigen into the inside of the left forearm of each participant. Retractable tuberculin-type syringes were used for each consenting participant. The same investigator using the same measuring instrument performed the intradermal tests and readings. Tests were read at 48/72 hours after injection and those that produced induration ≥ 5 mm in transverse diameter were considered to be histoplasmin positive [18]. Seventy two hours reading were for those who had skin test done on a Friday and had to return on Monday for reading.

#### Definition

High risk occupations were classified as agriculture/farming, builders, labourers, factory workers, and those who work with wood i.e. carpenters, furniture makers, wood cutters, wood sellers and artisans.

### Data analysis

Analyses were performed using SPSS Statistics v22.0 (IBM Corp., Armonk, NY, United States) and a 5% significance level was used throughout the study unless otherwise specified. Summary statistics were presented using frequencies and percentages or means, standard deviations and ranges, as appropriate. Chi-squared tests and Fisher’s exact tests were used to compare proportions between groups and independent samples t-tests were used to compare means. A 0.5% significance level was used to account for multiple testing in the single variable analysis. An exploratory logistic regression analysis, with skin sensitivity as the outcome, was used to assess whether any variables that were statistically significant in the single variable analysis were independently statistically significantly associated with skin sensitivity.

## Results

**Fig 1:**
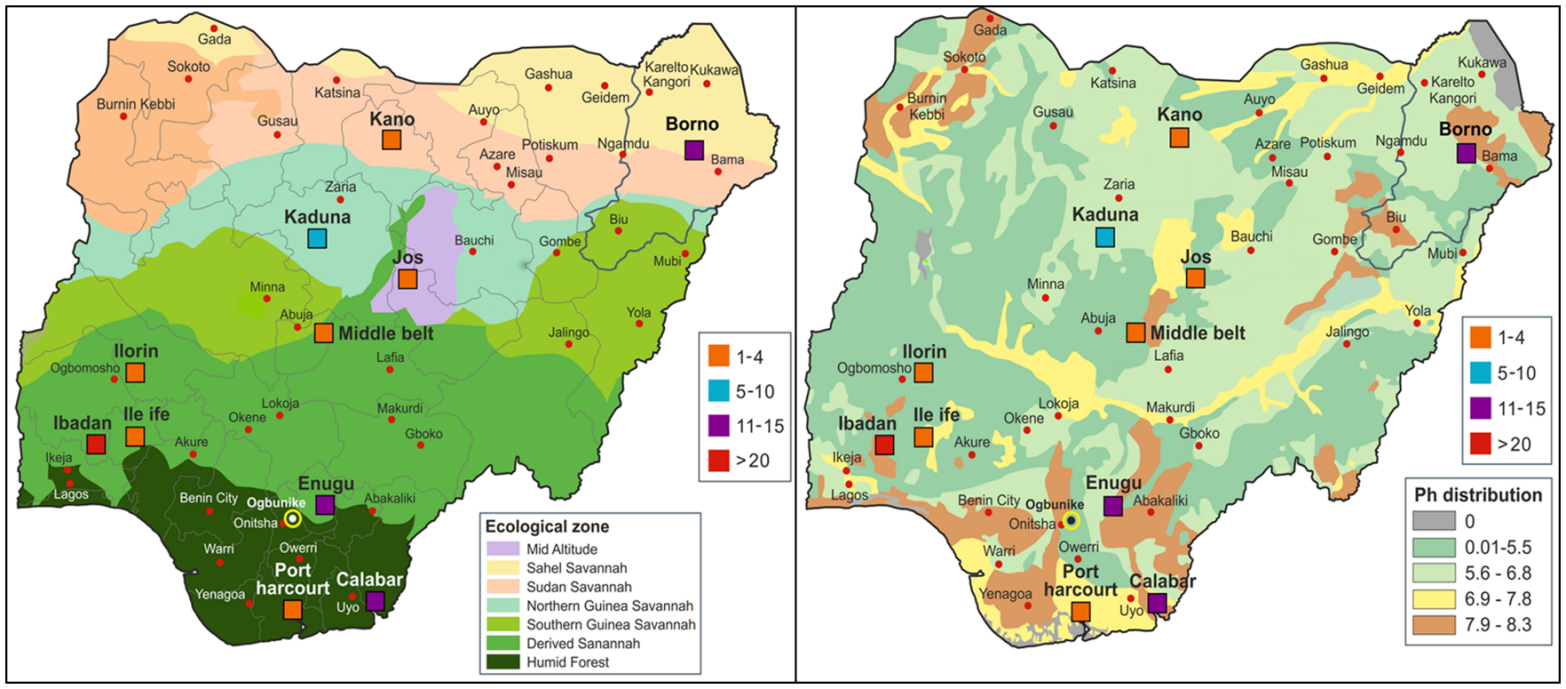
Distribution of reported cases of histoplasmosis in Nigeria in relation to vegetation (A) and soil types (B).

We identified 124 reported cases of histoplasmosis from Nigeria (see S1 File). However 4 cases of disseminated histoplasmosis were in immigrants in Europe and Asia, therefore we could not determine the states in Nigeria they came from; they were thus excluded. The remaining 120 cases of histoplasmosis reported from Nigeria were mapped (Fig 1) and compared to soil type and vegetation. All were cases of Hcd.

There were 750 participants across the 6 centres. Calabar was the only rural setting used for recruitment. The mean age of the participants was 39.2 years (SD 12.2) (range 14–85). Females (540 (72.0%)) outnumbered males (208 (27.7%)). Out of the 750 study participants, 467 (62.3%) were HIV negative, 247 (32.9%) were HIV positive and 36 (4.8%) had unknown HIV status. The average age (years) of HIV positive patients was 42.3 (SD 10.0) with a range of 18–72 years; the average age of HIV negative patients was 37.8 (SD 12.7) with a range of 14-85years. Thirty three percent (153/466) of the HIV negative patients were male, whereas 16% (39/246) of the HIV positive patients were male. While 173 (23%) had tertiary education, 53 (7%) had no formal education.

### Single variable analysis

A total of 32/735 (4.4%) participants (that returned for skin test reading) had a positive skin test, (Table 1). Study centre (p<0.001), education (p = 0.002) and age (p = 0.005) were significantly associated with positive skin reactivity at the 0.5% significance level (p≤0.005), while sex (p = 0.031) and occupation (p = 0.031) would have been significant at the 5% significance level—due to the issue of multiple testing the stricter 0.5% significance level was used. The highest positive rates were recorded from Benin City (13/86 (15%)) and Calabar (7/120 (6%)) while no positives were recorded in Lagos (p<0.001). Participants with a positive skin test had a higher mean age (45.1 +/- 14.6 years) compared to those without a positive skin test (39.0 +/- 11.9 years). The participants in Ibadan had the highest average age of 43.1 years (SD 10.4, range 19–72), while those in Benin City had the second highest average age (41.8 years, SD 16.6, range 18–85).

**Table 1:** Participants sociodemographic, clinical features and associated factors in relation to histoplasmin reactivity.

Variable	Reactive (≥5mm)	p-value
Study centre		<0.001^1^
Lagos	0/52 (0%)	
Yola	4/151 (3%)	
Ilorin	2/125 (2%)	
Calabar	7/120 (6%)	
Ibadan	6/201 (3%)	
Benin	13/86 (15%)	
Sex		0.031^1^
Female	17/527 (3%)	
Male	14/206 (7%)	
Highest education qualification		0.002^1^
None	7/50 (14%)	
Primary	7/181 (4%)	
Secondary	8/322 (2%)	
Tertiary	9/169 (5%)	
Thatched roof		0.59^2^
No	31/715 (4%)	
Yes	1/20 (5%)	
Corrugated roof		0.71^2^
No	1/46 (2%)	
Yes	31/688 (5%)	
Poultry within or around residence		0.73^1^
No	22/525 (4%)	
Yes	10/210 (5%)	
Warehouse (home/place of work)		>0.99^2^
No	12/402 (3%)	
Yes	1/46 (2%)	
Home/place of work in forested regions		0.51^2^
No	28/671 (4%)	
Yes	4/64 (6%)	
Contact with hunters		0.17^2^
No	22/579 (4%)	
Yes	3/35 (9%)	
Recent travel to areas with caves		0.67^2^
No	23/576 (4%)	
Yes	2/39 (5%)	
Living/working in areas with lots of birds		0.32^2^
No	4/107 (4%)	
Yes	0/44 (0%)	
House/workplace has orchard around it		0.012^2^
No	0/100 (0%)	
Yes	4/51 (8%)	
Heavy construction sites near workplace/home		>0.99^2^
No	31/698 (4%)	
Yes	1/37 (3%)	
Smoking		>0.99^2^
No	31/696 (4%)	
Yes	1/35 (3%)	
IV drug use		0.73^2^
No	28/661 (4%)	
Yes	3/58 (5%)	
Past chest infection		0.79^2^
No	29/638 (5%)	
Yes	3/89 (3%)	
HIV status		0.70^1^
Negative	18/457 (4%)	
Positive	11/242 (5%)	
Prior antifungal therapy		0.56^2^
No	25/473 (5%)	
Yes	2/76 (3%)	
Prior surgery		0.46^1^
No	28/603 (5%)	
Yes	4/126 (3%)	
Occupation		0.033^2^
Low risk	26/630 (4%)	
High risk	6/54 (11%)	

^1^Pearson chi-squared test

^2^Fisher’s exact test

Of all the risk factors analysed, lack of formal education, 7/50 (14%) (p = 0.002), those that lived/worked in areas with orchards (p = 0.012), 4/51 (8%), or in a high risk occupation (p = 0.031), 6/53 (11%), were more likely to be histoplasmin positive. Males had a higher rate of reactivity than females (p = 0.031, 7% vs 3%).

There did not appear to be a significant association with histoplasmin reactivity with other variables (Table 1).

### Multivariable analysis

Variables that were significant in the single variable analysis were included in the exploratory multivariable logistic regression. This is considered exploratory due to the relatively small number of participants with skin reactivity. The orchard variable was excluded despite being statistically significant due to the large amount of missing data. To find the variables that were independently statistically significantly associated with skin reactivity, the logistic regression model was run and variables that had a p-value greater than 0.05 were removed, one at time, until only variables with p<0.05 remained. Only study centre (p<0.001) and education (p = 0.002) were statistically significant in the final logistic regression model (see Table 2).

**Table 2:** Final logistic regression model.

Variable	Odds ratio for having skin reactivity (95% CI)	p-value
Study centre*		<0.001
Benin	1 (-)	
Calabar	0.47 (0.16–1.37)	0.17
Ibadan	0.21 (0.08–0.61)	0.004
Ilorin	0.07 (0.02–0.35)	0.001
Yola	0.12 (0.04–0.40)	0.001
Education		0.002
None	1 (-)	
Primary	0.17 (0.05–0.59)	0.005
Secondary	0.11 (0.03–0.36)	<0.001
Tertiary	0.32 (0.10–0.97)	0.045

*The category for Lagos, where no participant had skin reactivity, had to be removed as the logistic regression model could not converge to a solution with it included.

In an exploratory logistic regression, study centre remained statistically significant after accounting for sex, education, occupation and age, suggesting that these variables may not explain the higher rate of skin sensitivity in Benin.

## Discussion

Our finding of histoplasmin reactivity of 4.4% in the studied population is significantly lower than that of the last survey conducted three decades ago by Muotoe-Okafor and colleagues [18] which was 10.6% using 5mm as the cut-off. However their study was carried out in the vicinity of a natural focus of *Histoplasma capsulatum* var *duboisii*. In that study, participants included cave guides, traders and farmers examined in the immediate vicinity of the cave, 14/40 (35%) gave a positive skin test. Fifty-five out of 620 (8.8%) reacted positively to histoplasmin in another setting away from bat caves, although in the same state [18]. This location (Ogbunike, Anambra state) was not included in our study due to logistic challenges. Another plausible explanation is the fact that five out of our six study sites were in urban settings. However, our finding is similar to an earlier survey in Enugu state, South Eastern Nigeria, that gave a prevalence of 3.5% [7]. It was also not logistically feasible to include this site in our study. Only 16% of the HIV positive patients were male. Interestingly, more females are documented as HIV-infected in Nigeria population [21]. A possible explanation for this is the fact the ‘pick up’ rate of HIV in Nigeria is higher for women because of the screening policy for pregnant women (as the highest prevalence is in the reproductive age group), so they are more likely to start ART earlier than men, who more often present with AIDS defining infections. There is also the challenge of stigmatisation that prevents men from accessing screening programs. Socio-cultural and religious beliefs also hinder accessing screening. Moreover, more HIV-positive women consented to participate in this study.

Several environmental conditions have been documented to support the occurrence of *H*. *capsulatum* in an area [22]. These include high temperature, high humidity and soil pH above 10 and below 5 [22]. It is interesting to note that in present study, the soil type (pH ≥7) for the areas with high numbers of reported cases of histoplasmosis and high histoplasmin skin reactivity overlapped (Fig 1B). Benin City (15%) and Calabar (6%) which had the highest rates reported are both in the humid rain forest zone (Fig 1A). Participants with houses or workplaces adjacent to orchards had a higher rate of skin reactivity than those without fruit trees (8% vs 0%, p = 0.012). This result should be treated with caution due to the small number of participants who completed the specific question regarding living in the proximity of fruit trees (n = 151). The skin-test survey also revealed a significantly higher positivity rate among persons working/living around forests, majority of whom were from rural settings, this finding is consistent with a previous report that reported 8.8% histoplasmin reactivity in patients attending rural clinics. [18].

*H*. *capsulatum* is found in soil contaminated with bird or bat droppings; farmers, bird handlers, wood cutters, etc are at risk of of being exposed to *Histoplasma* and contracting histoplasmosis. Activities that disrupt the soil during excavation and the construction, demolition, and renovation of buildings generate infected dust; thus, construction workers in endemic areas should also be considered to be at a high risk of contracting histoplasmosis [23]. The level of education was statistically significant in our study and this is not surprising since most people with no formal or minimal formal education in Nigeria tend to do manual labour or become artisans which are part of high risk occupations for histoplasmosis.

Our findings demonstrated that study site was statistically significant (<0.001), the exploratory logistic regression suggested that differences in sex, education, occupation and age between the study centres may not explain the higher skin sensitivity rate in Benin City as study centre remained statistically significant after including these variables in the regression model. Benin City, though an urban city, has a significant number of individuals engaged in activities that are at high-risk for histoplasmosis such as- woodcraft, carving, bronze casting and terra cotta sculpture [24]. It is also famous for its forest and rubber plantations [25]. All these activities generate dust and if the soil is infected with *Histoplasma* then the workers are at risk. Interestingly, there have not been any case reports of histoplasmosis from this area possibly due to lack of skilled personnel and facilities to make this diagnosis. The rate of high-risk occupations, which appeared to be a risk factor for skin sensitivity, was second highest in Benin City (16%), Calabar had a 27% high risk occupation (predominately farmers) rate. The 5 study participants who worked with furniture or wood were all from Benin City and this is consistent with a report from Uganda that reported high histoplasmin reactivity amongst sawmill workers [9]. Lagos state, a cosmopolitan region, which is the commercial capital of Nigeria, had <2% histoplasmin skin reactivity. This is not surprising as none of the study participants were involved in the high risk occupations, however we did not explore if any had travelled to or spent significant time in endemic areas.

As expected, there was no significant association of HIV status with histoplasmin reactivity (p = 0.70) in our study, as we selected patients with higher CD4 counts. It is well documented that the greatest attributable risk factor for histoplasmosis is the spread of HIV [26], and thought to be about 100,000 cases annually [27]. Existing data demonstrates that in HIV-infected patients with histoplasmosis, the disease is disseminated in 95% of the cases, and in 90% of the cases it occurs in patients with CD4 counts below 200/mm3 [28]. A recent review showed that in Southern Africa; there were 119 cases of Hcc diagnosed with 80% (95) in HIV infected patients [15]. We suspect that there are missed cases of histoplasmosis in our Nigerian AIDS patients, which can be attributed to the lack of a rapid antigen test and facilities for prolonged fungal culture. By identifying localities of risk, we expect that focussed physician education programs and making rapid tests available would increase case identification.

Limitations of this study included exclusion of study participants that did not return for reading, however only 23 participants failed to return. Persons with previous history of histoplasmosis were also excluded because we did not want to skew the data.

In conclusion, we found 4.4% histoplasmin skin reactivity in a country with a high burden of tuberculosis and HIV/AIDS. Histoplasmosis is commonly misdiagnosed as tuberculosis. Interestingly, there have been no reports originating from Nigeria on histoplasmosis in the HIV community though it has been documented in Nigerian immigrants/refugees in Europe. There is dire need to re-strategize the management guidelines of this group of patients in Nigeria.

## Supporting information

S1 File Reported cases of histoplasmosis from Nigeria. (DOCX)

S1 Questionnaire Histoplasmin skin sensitivity survey questionaire. (DOCX)
