# Supplementary material for: Prior subclinical histoplasmosis revealed in Nigeria using histoplasmin skin testing
Source: PLoS One. 2018 May 9;13(5):e0196224. doi: 10.1371/journal.pone.0196224 (PMC5942784; doi:10.1371/journal.pone.0196224)
Supplement: S1 File — (DOCX) [file pone.0196224.s001.docx]

| **Nigerian City/Town** | **Type of histoplasmosis** | **HIV status** | **Reference** |
| --- | --- | --- | --- |
| Immigrant (Europe) | Disseminated histoplasmosis (3cases); Hcd | Positive | (Valero et al. 2017) |
|  |  |  |  |
| Immigrant (Saudi Arabia) | Disseminated histoplasmosis;Hcd | Positive | (Bashiri et al. 2016) |
| Ibadan | Histoplasmosis bone lesion; Hcd | Negative | (Daramola et al. 1979) |
| Enugu | Disseminated histoplasmosis in a child; Hcd | Negative | (Ubesie et al. 2013) |
| Enugu | Cutaneous histoplasmosis; Hcd | Negative | (Akpuaka et al. 1998) |
| Ilorin | Histoplasmosis lymphadenopathy; Hcd | Negative | (KA Adeniji 2000) |
| Middle belt | Subcutaneous histoplasmosis; Hcd | Negative | (Jacyk et al. 1981) |
| Ibadan | Cutaneous histoplasmosis(52 cases); Hcd | Negative | (LUCAS 1970) |
| Ibadan | Histoplasmosis, jenunal lesion; Hcd | Negative | (Adekunle et al. 1978) |
| Enugu | Histoplasmosis, bone lesions (6 Cases) in children; Hcd | Negative | (Onuigbo & Gugnani n.d.) |
| Ibadan | Histoplasmosis, vocal cord lesion | Negative | (Solanke et al. 1969) |
| Ibadan | Histoplasmosis, oral and bone lesions;Hcd | Negative | (Akinosi 1970) |
| Jos | Cutaneous histoplasmosis; Hcd | Negative | (Oguachuba & Gugnani 1982) |
| Borno | Histoplasmosis, oral lesions | Negative | (Olasoji et al. 1999) |
| Ile Ife | Histoplasmosis, bone lesions; Hcd | Negative | (Akinyoola et al. 2006) |
| Enugu | Histoplasmosis bone lesions; Hcd | Negative | (Onwuasoigwe & Gugnani n.d.) |
| Calabar | Histoplasmosis colonic mass; Hcd | Negative | (Khalil et al. 1989) |
| Calabar | Cutaneous histoplasmosis (12 cases); Hcd | Negative | (Khalil et al. 1999) |
| Kaduna | Histoplasmosis, bone lesion in child;Hcd | Negative | (Adekeye et al. 1988) |
| Enugu | Cutaneous and subcutaneous histoplasmosis (2cases); Hcd | Negative | (Egere et al. 1978) |
| Borno | Cutaneous and osteolytic hisplasmosis (2 adults and 2children); Hcd | Negative | (Khalil et al. 1998) |
| Ibadan | Subcutaneous histoplasmosis (3cases); Hcd | Negative | (Williams et al. 1971) |
| Kaduna | Histoplasmosis, oral lesion in a child; Hcd | Negative | (Mace 1978) |
| Ile Ife | Cutaneous and osteolytic histoplasmosis in a child: Hcd | Negative | (Shoroye & Oyedeji 1982) |
| Kano | Cutaneous and osteolytic hisplasmosis | Negative | (Uloko et al. 2012) |
| Ibadan | Disseminated histoplasmosis in 2 children | Negative | (Seriki et al. 1975) |
| Kaduna | Histoplasmosis bone lesion; Hcd | Negative | (Asamoa et al. 1990) |
| Kaduna | Cutaneous histoplasmosis (10 cases) | Negative | (Samaila & Abdullahi 2011) |
| Port harcourt | Histoplasmosis, bone lesion; Hcd | Negative | (Seleye-Fubara et al. 2011) |
| Ibadan | Pulmonary histoplasmosis (2cases); Hcd | Negative | (CLARK & GREENWOOD 1968) |
| Enugu | Histoplasmosis orbital cyst; Hcd | Negative | (Bansal et al. 1977) |
| Ibadan | Histoplasmosis orbital lesion in a child; Hcd | Negative | (Olurin et al. 1969) |
| Ile ife | Histoplasmosis, skull bone lesion: Hcd | Negative | (Ige et al. 1992) |

Distribution across the cities in Nigeria.

| Ibadan | 64 |
| --- | --- |
| Enugu | 14 |
| Calabar | 13 |
| Kaduna | 13 |
| Borno | 5 |
| Ile Ife | 3 |
| Ilorin | 3 |
| Jos | 1 |
| Kano | 1 |
| Middle belt | 2 |
| Port Harcourt | 1 |

**References**

Adekeye, E.O., Edwards, M.B. & Williams, H.K., 1988. Mandibular African histoplasmosis: Imitation of neoplasia or giant-cell granuloma? *Oral Surgery, Oral Medicine, Oral Pathology*, 65(1), pp.81–84. Available at: http://linkinghub.elsevier.com/retrieve/pii/0030422088901971 [Accessed August 17, 2017].

Adekunle, O.O., Sudhakaran, P. & Timeyin, E.D., 1978. African histoplasmosis of the jejunum. Report of a case. *The Journal of tropical medicine and hygiene*, 81(5), pp.88–90. Available at: http://www.ncbi.nlm.nih.gov/pubmed/660708.

Akinosi, J.O., 1970. African histoplasmosis presenting as a dental problem. *British Journal of Oral Surgery*, 8(1), pp.58–63. Available at: http://linkinghub.elsevier.com/retrieve/pii/S0007117X70800695 [Accessed August 17, 2017].

Akinyoola, A.L., Onayemi, O. & Famurewa, O.C., 2006. African histoplasmosis - masquerading as a malignant bone tumour. *Tropical Doctor*, 36(4), pp.250–251. Available at: http://www.ncbi.nlm.nih.gov/pubmed/17034712 [Accessed August 8, 2017].

Akpuaka, F.C., Gugnani, H.C. & Iregbulam, L.M., 1998. African histoplasmosis: report of two patients treated with amphotericin B and ketoconazole. *Mycoses*, 41(9–10), pp.363–364. Available at: http://doi.wiley.com/10.1111/j.1439-0507.1998.tb00354.x [Accessed August 8, 2017].

Asamoa, E.A. et al., 1990. Paediatric tumours of the jaws in northern Nigeria. *Journal of Cranio-Maxillofacial Surgery*, 18(3), pp.130–135. Available at: http://linkinghub.elsevier.com/retrieve/pii/S1010518205803300 [Accessed August 17, 2017].

Bansal, R.K., Suseelan, A.V. & Gugnani, H.C., 1977. Orbital cyst due to Histoplasma duboisii. *British Journal of Ophthalmology*, 61(1), pp.70–71.

Bashiri, S.A., Shahmirzadi, M.R.R. & Abro, A.H., 2016. A case of disseminated histoplasmosis which was misdiagnosed as squamous cell carcinoma of skin and pulmonary tuberculosis;case report, Rashid hospital,2013. In *Gulf Congress of Clinical Microbiology & Infectious Disease*. Dubai, United Arab Emirates, p. PP05.

CLARK, B.M. & GREENWOOD, B.M., 1968. Pulmonary lesions in African histoplasmosis. *Journal of Tropical Medicine and Hygiene*, 71(1), pp.4–10.

Daramola, J.O. et al., 1979. Maxillary african histoplasmosis mimicking malignant jaw tumour. *British Journal of Oral Surgery*, 16(3), pp.241–247. Available at: http://linkinghub.elsevier.com/retrieve/pii/0007117X79900301 [Accessed August 17, 2017].

Egere, J.U. et al., 1978. African histoplasmosis in Eastern Nigeria: report of two culturally proven cases treated with septrin and amphotericin B. *The Journal of tropical medicine and hygiene*, 81(11), pp.225–9. Available at: http://www.ncbi.nlm.nih.gov/pubmed/731765.

Ige, A.O., Nwosu, S.O. & Odesanmi, W.O., 1992. African histoplasmosis (Duboisii) of the skull with neurological complication--a case report and review of literature. *African journal of medicine and medical sciences*, 21(2), pp.19–21. Available at: http://www.ncbi.nlm.nih.gov/pubmed/1308076.

Jacyk, W.K., Lawande, R. V & Tulpule, S.S., 1981. Deep Mycoses in West Africa: A Report of 13 Cases and Review of the Nigerian Literature. *Journal of the National Medical Association*, 73(3), pp.251–259. Available at: http://www.ncbi.nlm.nih.gov/pmc/articles/PMC2609798/.

KA Adeniji, A.A., 2000. Peripheral lymphadenopathy in Nigeria. *African journal of medicine and medical sciences*, 29, pp.233–237.

Khalil, M. et al., 1999. Some deep mycoses diagnosed by histopathology in South Eastern Nigeria. *Revista iberoamericana de micologia*, 16(4), pp.221–4. Available at: http://www.ncbi.nlm.nih.gov/pubmed/18473552 [Accessed August 8, 2017].

Khalil, M.A., Hassan, A.W. & Gugnani, H.C., 1998. African histoplasmosis: report of four cases from northeastern Nigeria. *Mycoses*, 41(7–8), pp.293–295. Available at: http://doi.wiley.com/10.1111/j.1439-0507.1998.tb00341.x.

Khalil, M., Iwatt, A.R. & Gugnani, H.C., 1989. African histoplasmosis masquerading as carcinoma of the colon. *Diseases of the Colon & Rectum*, 32(6), pp.518–520. Available at: https://doi.org/10.1007/BF02554509.

LUCAS, A.O., 1970. CUTANEOUS MANIFESTATIONS OF AFRICAN HISTOPLASMOSIS ADETOKUNBO O. LUCAS. *British Journal of Dermatology*, 82(5), pp.435–447. Available at: http://doi.wiley.com/10.1111/j.1365-2133.1970.tb02203.x [Accessed August 13, 2017].

Mace, M.C., 1978. Oral African histoplasmosis resembling Burkitt’s lymphoma. *Oral Surgery, Oral Medicine, Oral Pathology*, 46(3), pp.407–412. Available at: http://linkinghub.elsevier.com/retrieve/pii/0030422078904061.

Oguachuba, H.N. & Gugnani, H.C., 1982. African histoplasmosis manifesting as a cutaneous tumour treated with econazole. *The Journal of tropical medicine and hygiene*, 85(6), pp.259–63. Available at: http://www.ncbi.nlm.nih.gov/pubmed/7154150 [Accessed August 13, 2017].

Olasoji, H.O., Pindiga, U.H. & Adeosun, O.O., 1999. African oral histoplasmosis mimicking lip carcinoma: case report. *East African medical journal*, 76(8), pp.475–6. Available at: http://www.ncbi.nlm.nih.gov/pubmed/10520359.

Olurin, O., Lucas, A.O. & Oyediran, A.B.O., 1969. Orbital Histoplasmosis Due to Histoplasma Duboisii. *American Journal of Ophthalmology*, 68(1), pp.14–18. Available at: http://linkinghub.elsevier.com/retrieve/pii/0002939469949290 [Accessed August 17, 2017].

Onuigbo, W.I. & Gugnani, H.C., Deep mycoses prevalent in the Igbos of Nigeria. *International journal of dermatology*, 15(6), pp.432–7. Available at: http://www.ncbi.nlm.nih.gov/pubmed/1279071 [Accessed August 13, 2017].

Onwuasoigwe, O. & Gugnani, H.C., African histoplasmosis: osteomyelitis of the radius. *Mycoses*, 41(3–4), pp.105–7. Available at: http://www.ncbi.nlm.nih.gov/pubmed/9670760 [Accessed August 8, 2017].

Samaila, M.O. & Abdullahi, K., 2011. Cutaneous manifestations of deep mycosis: an experience in a tropical pathology laboratory. *Indian journal of dermatology*, 56(3), pp.282–6. Available at: http://www.ncbi.nlm.nih.gov/pubmed/21772588 [Accessed August 8, 2017].

Seleye-Fubara, D., Etebu, E. & Bob-Yellowe, E., 2011. Granulomatous osteomyelitis: *A review of 13 cases in Port Harcourt, Nigeria*. *Sahel Medical Journal*, 14(1), pp.11–15. Available at: http://www.smjonline.org/article.asp?issn=1118-8561.

Seriki, O. et al., 1975. Disseminated histoplasmosis due to histoplasma capsulatum in two Nigerian children. *The Journal of tropical medicine and hygiene*, 78(12), pp.248–55. Available at: http://www.ncbi.nlm.nih.gov/pubmed/1223326 [Accessed August 8, 2017].

Shoroye, A. & Oyedeji, G.A., 1982. African histoplasmosis presenting as a facial tumour in a child. *Annals of Tropical Paediatrics*, 2(3), pp.147–149. Available at: http://www.tandfonline.com/doi/full/10.1080/02724936.1982.11748248.

Solanke, T.F., Akinyemi, O.O. & Clark, B.M., 1969. A case of histoplasmosis in a Nigerian. *The Journal of tropical medicine and hygiene*, 72(4), pp.101–4. Available at: http://www.ncbi.nlm.nih.gov/pubmed/5769716 [Accessed August 13, 2017].

Ubesie, A. et al., 2013. Disseminated Histoplasmosis in a 13-year-old girl: A case report. *African Health Sciences*, 13(2), pp.518–21. Available at: http://www.ncbi.nlm.nih.gov/pubmed/24235958 [Accessed August 8, 2017].

Uloko, A. et al., 2012. Histoplasmosis: An elusive re-emerging chest infection. *Nigerian Journal of Clinical Practice*, 15(2), p.235. Available at: http://www.ncbi.nlm.nih.gov/pubmed/22718181 [Accessed August 8, 2017].

Valero, C. et al., 2017. African histoplasmosis : new clinical and microbiological insights. , pp.1–9.

Williams, A.O., Lawson, E.A. & Lucas, A.O., 1971. African histoplasmosis due to Histoplasma duboisii. *Archives of pathology*, 92(5), pp.306–18. Available at: http://www.ncbi.nlm.nih.gov/pubmed/5165016.
