# Supplementary material for: Prior subclinical histoplasmosis revealed in Nigeria using histoplasmin skin testing
Source: PLoS One. 2018 May 9;13(5):e0196224. doi: 10.1371/journal.pone.0196224 (PMC5942784; doi:10.1371/journal.pone.0196224)
Supplement: S1 Questionnaire — (DOCX) [file pone.0196224.s002.docx]

**Sociomedical historyHistoplasmin skin sensitivity survey**

Date: Serial no:

Age: Sex: Marital status:

Occupation:

Highest education qualification: Pri 2^0^  3^0^

Home address (general area and town)

Pls tick appropriate answers below:

1. Thatched roof house
2. Corrugated roof house
3. Poultry within or around residence
4. Warehouse (home/ place of work)
5. Home or place of work in forested regions
6. Contact with hunters in the past
7. History of travel to areas with caves
8. History of living or working in areas with lots of birds
9. Your house of workplace has lots of fruit trees around it.
10. Heavy construction sites near work place or home

Smoking? Yes No

Past Medical History:

Have you now or in the past self injected non-medically prescribed drugs?: yes no

Past chest infection: yes no

If yes, duration: less than 3months more than 6months

Have been diagnosed with HIV infection?: positive negative date:

If yes what was your last CD4 count date:

Prior antifungal therapy (fluconazole): dates:

If you received this drug, what was the condition that you were suffering from?Indication for fluconazole therapy:

Prior surgery (s)?: yes no date(s):

Skin reading results: mm
